# Activated Carbon Modified with Copper for Adsorption of Propanethiol

**DOI:** 10.3390/ijms11030927

**Published:** 2010-03-04

**Authors:** Juan Carlos Moreno-Piraján, Joaquín Tirano, Brisa Salamanca, Liliana Giraldo

**Affiliations:** 1 Grupo de Investigación en Sólidos Porosos y Calorimetría, Departamento de Química, Facultad de Ciencias, Universidad de los Andes, Carrera 1 No. 18 A 10, Bogotá, Colombia; 2 Departamento de Ingeniería Química, Facultad de Ingeniería, Universidad de los Andes, Carrera 1 No. 18 A 10, Bogotá, Colombia; 3 Departamento de Química, Facultad de Ciencias, Universidad Nacional de Colombia, Carrera 30 No. 43-00, Bogotá, Colombia

**Keywords:** activated carbon, propanethiol, adsorption isotherms, kinetics, immersion calorimetry, decomposition mechanism

## Abstract

Activated carbons were characterized texturally and chemically before and after treatment, using surface area determination in the BET model, Boehm titration, TPR, DRX and immersion calorimetry. The adsorption capacity and the kinetics of sulphur compound removal were determined by gas chromatography. It was established that the propanethiol retention capacity is dependent on the number of oxygenated groups generated on the activated carbon surface and that activated carbon modified with CuO at 0.25 M shows the highest retention of propanethiol. Additionally is proposed a mechanism of decomposition of propenothiol with carbon-copper system.

## 1. Introduction

Chemical compounds containing the mercaptan group (-SH) are highly odorous and volatile, and even small amounts of such species in the air, for example CH_3_SH (minimum odour threshold: about 5 ppm), can make us uncomfortable. The removal of -SH species from the air is important for our comfort, and activated carbons are widely used as one of the most effective adsorbents [1–3]. In general, the adsorption of organic compounds by activated carbon is largely influenced by characteristics such as pore size, distribution, morphology and surface properties. Propanethiol is physically or chemically adsorbed onto activated carbon. Since the propanethiol molecule is of average size, the surface properties and pore characteristics of the activated carbon are thought to play an important role in its adsorption. On the other hand, it has been reported that the oxidation of methyl mercaptan to dimethyl disulphide takes place at the surface of activated carbon containing functional groups such as carboxyl group in dry/wet air [4–6]. In addition, there are a few reports on the adsorption/oxidation of mercaptans on surface-modified activated carbon [7–9]. However, there are few reports discussing the real adsorption of propanethiol on activated carbon.

In reference to the surface modification of activated carbon, the introduction of carboxyl and amino groups, and the preparation of functional group-free activated carbon, have been reported [10–17]. The introduction of carboxyl groups onto the surface of activated carbon by HNO_3_ or H_2_O_2_ treatments has been investigated [10–13]. The introduction of amino groups has been carried out by various methods, e.g., reduction of the nitro group formed by a HNO_3_/H_2_SO_4_ mixture [14–16], and treatment with gaseous ammonia [17]. On the other hand, most functional groups generally vanish with heat-treatment under Ar or N_2_. Copper oxide adsorbents are normally quite unreactive with thiophenic sulphur compounds (e.g., propanethiol), while in contrast, they readily react with and adsorb mercaptans (also known as thiols). It is well known that Cu-based adsorbents are less efficient than those that are Ni-based, and sulphur is often observed to leak from this type of guard bed. However, thiols act in a very different manner with copper oxide, as an irreversible redox reaction takes place, converting the thiol and Cu^2+^ into copper(I) thiolate in a two-step process [18]:

(1) $$\text{Step}\;1:\;2\text{RSH}+2\text{CuO}\to \text{RSSR}+{\text{Cu}}_{2}\text{O}+{\text{H}}_{2}\text{O}$$

(2) $$\text{Step}\;2:\;2\text{RSH}+{\text{Cu}}_{2}\text{O}\to 2\text{RSCu}+{\text{H}}_{2}\text{O}$$

In the first step, reduction/oxidation occurs between the thiol and CuO to form a disulphide and copper(I) oxide. The reaction continues as the thiol reacts with the surface copper(I) oxide to form copper(I) thiolate. Similar chemistry occurs between the surface of the copper metal and thiol, according to the following reaction, in which the copper metal serves as a reducing agent to liberate H_2_:

(3) $$2\text{RSH}+2\text{Cu}\to 2\text{RSCu}+{\text{H}}_{2}$$

**Figure 1.**
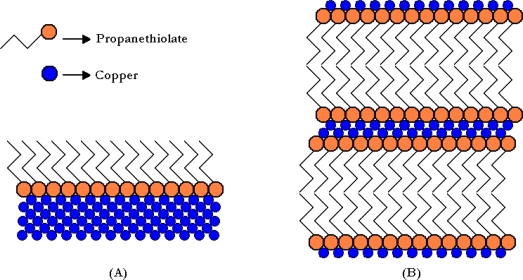
Simple representations of (A) self-assembled monolayer (SAM) of propanethiol on the surface of copper metal, and (B) self-assembled multilayers of copper(I) propanethiolate.

It is well known that thiols will self-assemble into strictly arranged monolayers (commonly known as SAMs) onto the surface of coinage metals, especially gold, silver, and copper [18]. These SAMs have been intensively studied and are of great interest due to the unique properties of the resulting surfaces, including stabilization and passivation to electrochemistry and other reactions. While much of the study of copper/thiol chemistry has been restricted to the formation and properties of SAMs, it has been shown that multilayers can form into independent lamellar structures (Figure 1) [10–21]. It was discovered in this study that under the right conditions, these types of layered compounds can be formed in a sulphur guard bed at elevated temperature and pressure, and in the presence of a hydrocarbon solvent. The redox chemistry of Cu^2+^ and mercaptans has long been known, and in fact a process to sweeten gasoline has been used since the early 20th century based upon this chemistry [22].

In the so-called “Perco” process, copper(II) chloride is placed in an air percolating bed with gasoline, and the foul smelling mercaptans are converted into the less offensive disulphides. In this case, the Cu^2+^ is not consumed in the process because the oxygen from the air percolation reoxidizes the partially reduced Cu^+^ back to Cu^2+^, according to the following reaction.

Perco sweetening process:

(4) $$\text{Step}\;1:\;2\text{HSR}+2{\text{Cu}}^{2+}\to \text{RSSR}+2{\text{Cu}}^{+}+2{\text{H}}^{+}$$

(5) $$\text{Step}\;2:\;2{\text{Cu}}^{+}1/2{\text{O}}_{2}+2{\text{H}}^{+}\to 2{\text{Cu}}^{+}+{\text{H}}_{2}\text{O}$$

(6) $$\text{net}\;\text{reaction}:\;2\text{HSR}+1/2{\text{O}}_{2}\to \text{RSSR}+{\text{H}}_{2}\text{O}$$

Notice that in the Perco sweetening process, the copper is never allowed to form the stable copper(I) thiolate compound. Finally, the routine synthesis of copper(I) thiolate, such as that made from propanethiol, is carried out using mixed aqueous and organic phases, where the aqueous phase contains a Cu^2+^ salt and the organic phase contains the thiol. These procedures vary and have been reported previously [20], but the general procedure only illustrates the common method of synthesizing copper (I) thiolate crystals. What is shown in the present work is that these layered materials may also be formed in conditions similar to those used in sulphur trap beds in refineries.

Against this background, in this work we investigated the influence of the surface modification of activated carbon by acid-treatment for different times and concentrations, and by the impregnation with copper, on the adsorption of propanethiol from an aqueous solution. A study of the kinetic reaction was also carried out, and based on the results and a review of the literature; we explain the role of copper in the decomposition of propanethiol.

## 2. Results and Discussion

### 2.1. Characterization

#### 2.1.1. N_2_ adsorption isotherms

**Figure 2.**
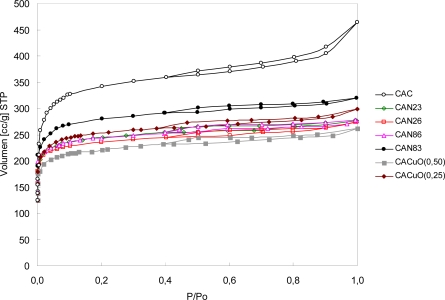
N_2_ adsorption isotherms at 77 K.

In order to investigate the influence on the textural properties of the surface of activated carbons, isotherms of adsorption–desorption of N_2_ at 77 K were determined and are shown in Figure 2.

The isotherms were type I according to the IUPAC classification. The major consumption of N_2_ in the adsorption–desorption isotherms of the activated carbons occurred at relatively low relative pressure (<0.2) and reached a plateau at high relative pressure. These results indicate that the activated carbons obtained by the various treatments were microporous. In the case of activated carbon treated with acid, the amounts of N_2_ adsorbed decreased with an increasing concentration of HNO_3_, as in those treated with CuO, probably because this treatment produced highly blocked pores in the activated carbon. With regards CAN83, there was a decrease in its area but not to the same magnitude as the other carbons. In the case of treatment with HNO_3_ for 8 h, the surface area of the activated carbon was still considerable.

The CuO/CAC catalysts that were prepared by impregnation of the pre-treated CAC with an aqueous solution of the precursor (copper nitrate, Cu(NO_3_)_2_ · 3H_2_O) showed a decrease in surface area, but the volume of micro and meso pores was of the same order of magnitude as the other porous materials that were synthesized.

The pore characteristics of activated carbons based on N_2_ adsorption–desorption isotherms are shown in Table 1.

The CAC was highly microporous and had a high BET surface area of more than 1400 m^2^/g. In the case of acid-treated activated carbons, the BET surface area decreased following treatment with HNO_3_ solutions. It is assumed that the porous structure of activated carbon is destroyed partially by the strong oxidative effect of HNO_3_ solutions and that micropores are not detected. Table 1 reports the surface area calculated by the BET method, the total volume, and the mesoporous and microporous volume of each activated carbon.

#### 2.1.2. Boehm titration

The numbers of acidic groups on the activated carbons as estimated by a titration method [13] are shown in Table 2. The treatment of a carbon surface with HNO_3_ solutions results in an increase in the amount of oxygented groups, both acidic and basic, on that surface. A higher number of oxygenated groups were found on CAC compared with the other activated carbons obtained in this work, which shows the effect of the oxidizing agent HNO_3_ as reported in the literature.

Table 2 shows that in the activated carbons, the amounts of oxygenated groups resulting from the HNO_3_ treatment increases with time, and a similar behaviour is observed after treatment with copper ions as the amount of oxygenated groups also increases. This amount of copper deposited on the carbon surface is greater, and a corresponding enhanced catalytic effect of these ions in the decomposition of propanethiol may be expected.

The number of oxygenated groups in each of the samples of activated carbon decreased, as shown in Table 2. The basicity of the activated carbons modified with nitric acid was less than that in CAC in all cases. The activated carbons modified with copper nitrate showed higher acidity since copper has a higher affinity with the base, consuming more moles of sodium hydroxide. The total acidity increased with the increasing number of oxygenated groups.

#### 2.1.3. Immersion calorimetry

The results on the immersion enthalpy of the activated carbons in water and propanethiol are shown in Table 3. The surface chemistry is one of the variables that most affects the magnitude of the heat generated by carbon immersed in the test liquids [13]. High values of immersion enthalpies of activated carbon in propanethiol were obtained for CACuO (0.25) and CACuO (0.5). The modified activated carbons did not show a significant interaction because the thermal effect that develops when the carbons are immersed in propanethiol is low; the enthalpy of the samples increased without increasing the concentration of the activating agent or the duration of modification. Considering the order of magnitude of the enthalpic values of the adsorbent–adsorbate interaction, this may be considered a physiorption [18].

This is because in this case there is an interaction of a chemical, when comparing the orders of magnitude between the enthalpy values is obtained in propanethiol values are higher compared to water. In addition, we see the effects the different treatments have on the activated carbon, which can be characterized by immersion microcalorimetry.

#### 2.1.4. X-ray diffraction

**Figure 3.**
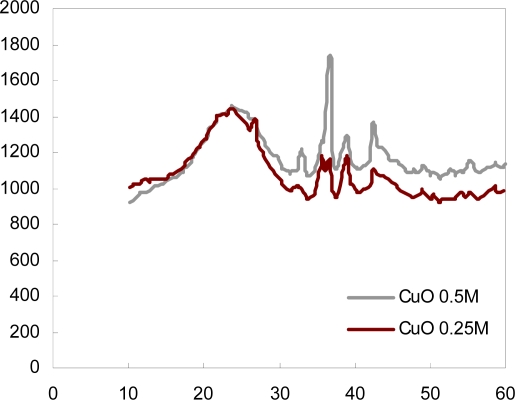
X-ray diffraction for the activated carbons impregnated with copper oxide.

Figure 3 shows the XRD patterns of the support catalysts CACCu(0.25) and CACCu(0.5). The XRD pattern for copper oxide has peaks at angles of 35.7 and 38.55 of 2θ; these angles of reflection indicate the formation and presence of copper oxide particles on activated carbon and are consistent with the literature [13]. Figure 2 shows these peaks for CACuO(0.25) and CACuO(0.50).

Analysis of the spent adsorbent material by powder X-ray diffraction revealed very strong reflections with the preferred orientation of copper(I) propanethiolate crystals (Figure 2). The strongest intensity was measured in CACCu(0.5) before and after top adsorption and decomposition of propanethiol, indicating the formation of relatively large crystals. Similar diffraction patterns are shown for other thiolate crystals in reference [20], but with what seem to be much lower intensities, confirming the redox chemistry between Cu^2+^ and the propanethiol in the solutions in hexane.

Based on Bragg’s law of diffraction and the peak positions for the crystals, the interlayer spacing for the layered copper(I) propanethiolate crystals was 35.7° and 38.55°, which is in close agreement with the values reported in the literature.

**Figure 4.**
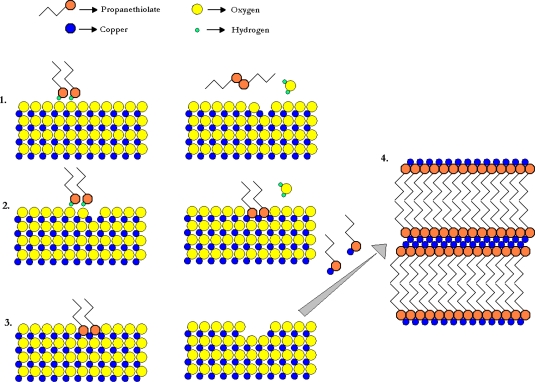
Proposed reaction mechanism occurring at 298 K and 1 bar between CACCu and propanethiol to form crystals of copper(I) propanethiolate. (1) Reduction/oxidation to form a disulphide and Cu_2_O. (2) Displacement to form a self-assembled monolayer of propanethiol on the surface of the CuO. (3) Dissolution of the copper(I) propanethiolate to expose fresh surface of the material. (4) Crystallization of self-assembled multilayers of copper(I) propanethiolate.

Therefore, the conclusion is that in the bulk of the copper is utilized in this process. As already shown, the dispersion of the copper particles enhances the total capacity for sulphur adsorption. In light of the above observations and comparing these data with the chemical mechanisms that were reviewed in the Introduction, Figure 4 illustrates the proposed primary mechanism for the adsorption-decomposition of mercaptans using the activated carbon-copper as catalyst, based on the proposal by Turbeville *et al*. [24].

**Figure 5.**
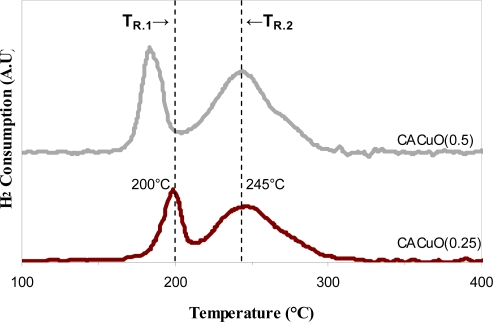
Temperature programmed reduction.

Figure 5 is provided as an illustration of a proposed primary mechanism for adsorption of mercaptans onto copper-containing adsorbents in these conditions. First, the well-known redox chemistry occurs between the CuO and mercaptan, resulting in a disulphide and a Cu_2_O site on the surface. This is followed by a displacement reaction of the Cu_2_O with the mercaptan, in this case, forming copper(I) butanethiolate on the surface. It should be noted that at this point, a SAM has effectively been formed on the surface. Indeed, SAM formation on the surface of CuO has been reported to occur [2,9]. Since copper(I) propanethiolate crystals were formed separately from the adsorbent materials, it is necessary for the surface species to dissolve into the feed liquid stream and migrate to crystallize at another site. This mechanism is therefore quite effective in uncovering bulk CuO in the material, making it available to participate in the adsorption of the mercaptan.

#### 2.1.5. Temperature programmed reduction (TPR)

The reducibility of the synthesized catalysts was evaluated by TPR measurements in a H_2_ flow, and the results are shown in Figure 5. The main reactions showed two peaks in the temperature range from 150 to 300 °C. These peaks were interpreted as corresponding to the stepwise reduction of CuO [15]. The peak at the lower temperature corresponds to the reduction of Cu^2+^ to Cu^+^ and the peak at the relatively high temperature corresponds to the reduction of Cu^+^ to Cu^0^ [17]. In the case of CACCu(0.5), the first peak was observed at a lower temperature (185 °C, TR_1_) than that of the CACCuO(0.50) catalyst (197 °C), because the change in the oxygenated content promotes hydrogen consumption at a lower temperature range. Similarly, the introduction of more oxygenated groups induces a shift in the first and second peaks towards a lower temperature. These peak shifts indicate that the reduction of CuO to Cu occurred more easily in the CACuO(0.25) and CANCu(0.50) catalysts because of the development of acid and basic groups that increase oxygen migration and permit the oxygen emitted from CuO to migrate more actively to the catalyst surface.

Figure 5 shows that the activated carbon with the highest quantity of Cu is the CACuO(0.5), which shows a higher intensity than the activated carbon modified with 0.25 M copper nitrate.

### 2.2. Adsorption isotherms

#### 2.2.1. Propanethiol adsorption isotherms

**Figure 6.**
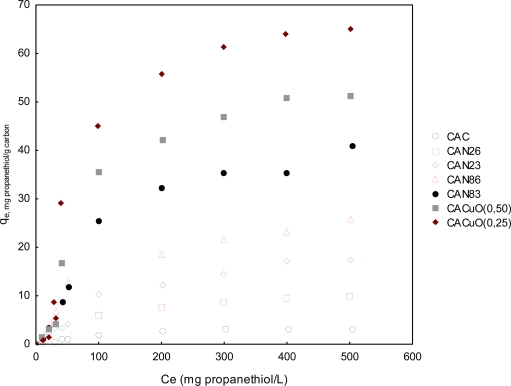
Propanethiol adsorption isotherms.

Figure 6 shows the adsorption of propanethiol on the activated carbons as a function of initial concentration.

The amounts of propanethiol adsorbed on the original activated carbon (AC) increased with time and reached a plateau, as shown. Propanethiol adsorbs physically, e.g., by a dispersive force, on the surface of acid-metal-treated activated carbon. The amounts of propanethiol adsorbed on treated activated carbon also increased with time. In the case of acid-treated activated carbons, the adsorption of propanethiol increased with the concentration of HNO_3_ solutions used for treatment, more so than in those treated with copper.Since the number of acidic groups on activated carbons increased with the concentration of HNO_3_ solutions, the amount of propanethiol adsorbed must be influenced by the acidic groups. With regards the effect of the carboxyl group, the catalytic effect on the oxidation of methyl mercaptan to dimethyl disulphide by oxygen in air under dry and wet conditions has previously been reported, with values consistent with those obtained in this work. Therefore, other factors, e.g., hydrogen bonding between carboxyl groups and thiol groups of methyl mercaptan rather than oxidation of methyl mercaptan, are thought to play a role in the increase in the adsorption of propanethiol.

#### 2.2.2. Freundlich isotherm

**Figure 7.**
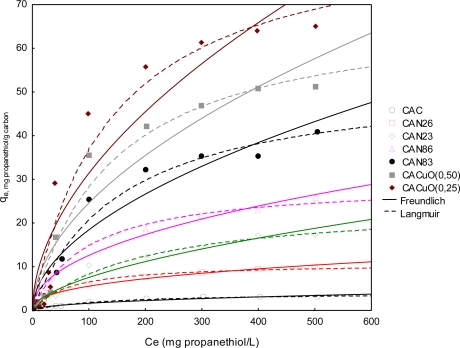
Adsorption isotherm: Langmuir and Freundlich models.

Table 4 shows the adsorption constants obtained when applying the Freundlich model to the experimental data [8,9]; the K_F_ values for the CAN86 and CACuO(0.25) samples (6.63 and 7.21, respectively) display the highest values compared to the other samples, indicating that the adsorption capacity higher for these samples. When the correlation coefficients are analyzed, it is observed that they fall in a range between 0.993–0.879, suggesting a high dispersion and indicating that this model does not adequately describe the adsorption of propanethiol from the hexane solution onto the solids. Figure 7 shows the dotted lines of the Freundlich model.

#### 2.2.3. Langmuir isotherm

Table 5 shows adjustment of the experimental data to fit the Langmuir model [8,9]. The adsorption constants and R^2^ show a reduced range of 0.996–0.982, which indicate that this model was a better fit for the experimental data obtained in the isotherms.

The higher values for the maximum adsorbed quantity found for the samples of activated carbon impregnated with copper agree with the results obtained for the immersion enthalpies; the K_L_ constant values indicated the high adsorption of the propanethiol in these samples. With regards the samples modified by surface oxidation, CAN26 showed the highest adsorption value, being the sample that was modified with 6 M nitric acid solution, and thus showing the influence of the superficial chemistry on propanethiol adsorption. Figure 7 shows the fit to this model with solid lines; it was found that this model was a better fit and therefore adequately describes the adsorption of propanethiol on activated carbon.

### 2.3. Adsorption kinetics

#### 2.3.1. Adsorption of propanethiol as a function of time

**Figure 8.**
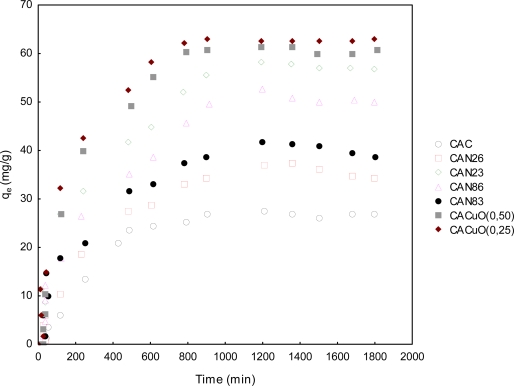
Kinetics of propanethiol removal.

Two kinetic models were studied, the pseudo first and pseudo second order [8,9,14]. Figure 8 shows the experimental results obtained for the solute adsorbed as a function of time on the surface of the activated carbon, which becomes saturated after about 800 minutes. The CACuO(0.25) sample showed the highest adsorption capacity for propanethiol and the original activated carbon the lowest (36%).

#### 2.3.2. Pseudo first order kinetics

Table 6 shows the quantity of propanethiol adsorbed at equilibrium for each of the activated carbon samples synthesized, using the pseudo first order model, the constant kinetics and a factor of correlation R^2^ of between 0.982–0.963. These values suggest dispersion of the experimental data.

#### 2.3.3. Pseudo second order kinetics

The pseudo second order model has a better fit for the CAN than the CACuO. The correlation coefficients, between 0.997–0.983 are better than those of the Freundlich model. Table 7 shows the amount of propanethiol adsorbed in equilibrium, the velocity constant of the model and the correlation factors; these values are taken from the linearization of the model.

By comparing with the previous kinetic model it is possible to establish that the kinetics of propanethiol removal to a second-rate kinetics, since this model adjusts the experimental data with less dispersion.

## 3. Experimental Section

### 3.1. Materials

Activated carbon (**CAC**) prepared from a commercial, coconut shell-derived carbon source through steam physical activation at about 800 °C was used as the catalyst support in this study. Before use, the AC support was pre-treated with nitric acid solutions at concentrations of 3 and 6 M in order to carry out the chemical modification. Soxhlet equipment was used for this procedure, and 150 mL of the solutions containing 20 g of activated carbon were added and heated for 2 to 8 h. The activated carbon was washed with hot water until it reached a constant pH of 6.5 after modification. The acid-treated supports were designated as follows: (a) acid-treated activated carbon (**CAN23**): CAC was treated with HNO_3_ 2 M for 2 h; (b) acid-treated activated carbon (**CAN26**): CAC was treated with HNO_3_ 2 M for 6 h; (c) acid-treated activated carbon (**CAN83**): CAC was treated with HNO_3_ 8 M for 3 h; (d) acid-treated activated carbon (**CAN86**): CAC was treated with HNO_3_ 8 M for 6 h.

The CuO/CAC catalysts were prepared by impregnation of the pre-treated CAC with an aqueous solution of the precursor (copper nitrate, Cu(NO_3_)_2_·3H_2_O) at the appropriate concentration (0.25 M and 0.5 M) to obtain Cu loadings of about 3 wt.%. During impregnation, the solutions were heated at 70 °C with constant stirring until the liquids were totally eliminated. Then, the catalysts were dried at 110 °C for 2 h and subsequently calcined in flowing air at 450 °C for 2 h. The approximate weight loss of CAC-supported catalysts during the calcination step at 450 °C was about 20%. The initial amounts of CuO coated were estimated from the initial amounts of support and Cu(NO_3_)_2_·3H_2_O. The copper-treated supports were designated as follows: (e) acid-treated activated carbon (**CACuO(0.25)**): CAC was treated with CuNO_3_ 0.25 M; (f) acid-treated activated carbon (**CACuO(0.5)**): CAC was treated with CuNO_3_ 0.5 M.

### 3.2. Sample characterization

#### 3.2.1. Nitrogen adsorption isotherm at 77 K

N_2_ adsorption isotherms at 77 K were measured in a volumetric system (Autosorb-3B, Quantachrome). For the adsorption data analysis the Dubinin–Radushkevich equation (DR) was used. The activated carbon samples, about 0.100 g, were degassed at 523 K for 3 h in an Autosorb 3B (Quantachrome Co.). The corresponding adsorption nitrogen isotherms were obtained in this apparatus at 77 K [5].

#### 3.2.2. Boehm titration

The basic and acid sites of the activated carbons were determined by means of the titration method proposed by Boehm using 0.1 M HCl, NaHCO_3,_ Na_2_CO_3_ and NaOH solutions [6]. In this method, 50 mL of the solution is placed in contact with 0.500 g of activated carbon in a glass flask for 48 h at a constant temperature of 298 K. A sample of 10 mL was titrated with the corresponding 0.1 M solution. Titration was carried out using a Trito Line Alpha Plus (SCHOTT Instruments, Woburn, MA, USA) [4].

#### 3.2.3. Immersion calorimetry

Determinations of the immersion enthalpy of the activated carbons were carried out in water and propanethiol. A Calvet-type heat conduction microcalorimeter of local construction [23] was used in order to determine the immersion heat. The cell was filled with 8 mL of the liquid. A sample of activated carbon weighing approximately 0.100 g was placed in the calorimetric cell inside a glass ampoule. When the equipment reached an equilibrium temperature the glass ampoule was broken; the thermal effect generated was recorded and electrically calibrated.

#### 3.2.4. X-ray diffraction

The activated carbon samples were macerated to obtain a fine powder prior to measurements made using this technique. The powder was placed in a support to measure the X-ray spectrum, with data taken from 2 to 180° at intervals of 0.02°/min. Rigaku Mini Flex equipment was used to take the measurements.

#### 3.2.5. Temperature programmed reduction (TPR)

The activated carbon samples were submitted to a hydrogen flow of 50 mL per minute. The temperature was increased gradually, in order to determine the temperature at which reduction took place. A MICROMERITICS 2720 chemisortometer with a mixture of He/H_2_ (10:90) was used.

### 3.3. Equilibrium adsorption isotherm

The method used to obtain the equilibrium isotherm was the batch type system. A sample of activated carbon weighing 1,000 g was placed in 250 mL of propanethiol in *n*-hexane solutions (30–500 ppm) in an Erlenmeyer flask. The solution was stirred and kept at a constant temperature to achieve equilibrium. An aliquot of 1 mL of each sample was analysed by gas chromatography using a Shimadzu QP2010 Plus GC-MS equipped with a flame ionization detector [9,10].

### 3.4. Kinetics of adsorption

Experiments performed to establish the kinetics of propanethiol removal by the activated carbons were carried out at room temperature and atmospheric pressure. The solids were previously dried to 100 °C in a furnace for 24 h and then weighed, and approximately 1,000 g of each was placed in 150 mL of propanethiol solution of 500 ppm in hexane and transferred into an Erlenmeyer flask, which was then sealed and sampled periodically. The samples were analysed by gas chromatography [9].

## 4. Conclusions

The surface chemistry and textural properties of the commercial AC changed when it was modified by means of nitric acid and Cu(NO_3_)_2_ solutions at different concentrations. The presence of CuO on the surface of the AC corresponded to impregnation with copper salt, as established by Temperature Programmed Reduction and X-ray diffraction. Propanethiol was adsorbed by the modified carbons depending on the amount of oxygenated groups on the surface of the activated carbons. The activated carbon showing the highest propanethiol retention was obtained with 0.25M copper nitrate solution. It was demonstrated that by modifying the chemistry and texture of the AC, propanethiol adsorption can be increased by 84%. For the AC modified by nitric acid solution the most important factor is the concentration.

## Figures and Tables

**Table 1.** Surface area (S_BET_) and total pore volume (V_t_) from the N_2_ adsorption isotherms at 77 K.

**Carbon**	**S_BET_ (m^2^/g)**	**V_t_ (cm^3^/g)**	**V_mic_ (cm^3^/g)**	**V_meso_ (cm^3^/g)**
CAC	1449	0.66	0.43	0.23
CAN23	884	0.39	0.33	0.06
CAN26	855	0.39	0.32	0.07
CAN83	1039	0.46	0.37	0.09
CAN86	875	0.36	0.33	0.03
CACuO(0.50)	872	0.41	0.34	0.07
CACuO(0.25)	992	0.36	0.30	0.06

**Table 2.** Boehm titration results and superficial chemistry of the AC.

**AC**	Carboxylates*	Lactones*	Phenols*	**AC**	Oxygenated total *	Total acidity*	Total basicity*
CAC	2.14	2.37	83.2	CACuO(0.25)	281	281	86.8
CAN23	61.3	65.7	108.5	CACuO(0.5)	282	279	60.3
CAN26	62.4	60.8	133.7	CAN26	301	256	14.2
CAN83	63.5	118.6	140.6	CAN86	328	315	5.82
CAN86	65.1	120.4	142.3	CAN83	330	320	2.26
CACuO(0.25)	3.51	140	135	CAN83	164	163	24.0
CACuO(0.5)	32.9	234	13.7	CAC	88	87	56.9

* in (μmolg^−1^).

**Table 3.** Immersion enthalpies of activated carbons in water and propanethiol.

**Carbon**	**−ΔH_imm_Water**	**−ΔH_imm_Propanethiol**
CAC	25.55	35.21
CAN23	23.10	31.72
CAN26	10.82	38.70
CAN83	23.89	33.22
CAN86	18.03	64.40
CANCu(0.5)	10.94	113.34
CANCuO(0.25)	25.58	138.47

**Table 4.** Freundlich model constants.

**Carbon**	**K_F_ (mg/g) (L/mg)^1/n^**	**n**	**R^2^**
CAC	0.47	3.12	0.972
CAN83	1.11	2.45	0.955
CAN86	6.63	5.41	0.993
CAN26	3.19	2.87	0.879
CAN23	3.62	6.33	0.918
CACuO(0.50)	5.38	2.78	0.974
CACuO(0.25)	7.21	3.16	0.952

**Table 5.** Langmuir model constants.

**Carbon**	**q_m_ (mg/g)**	**K_L_ (L/mg)**	**R^2^**
CAC	3.731	0.014	0.996
CAN83	14.706	0.013	0.982
CAN86	21.739	0.031	0.990
CAN26	27.027	0.021	0.983
CAN23	10.870	0.021	0.997
CACuO(0.50)	40.000	0.066	0.997
CACuO(0.25)	43.478	0.085	0.995

**Table 6.** Constants kinetics for a pseudo first order reaction.

**Carbon**	**q_e_ (mg/g)**	**k_1_ (min^−1^)**	**R^2^**
CAC	28.50	0.003	0.982
CAN23	35.59	0.003	0.984
CAN26	40.29	0.003	0.963
CAN83	48.47	0.002	0.971
CAN86	54.27	0.002	0.982
CACuO(0.50)	57.86	0.004	0.975
CACuO(0.25)	58.15	0.003	0.982

**Table 7.** Pseudo second order model constants.

**Carbon**	**q_e_ (mg/g)**	**k_2_ (g/mg.min)**	**R^2^**
CAC	33.33	0.00010353	0.997
CAN23	41.67	9.7166E-05	0.983
CAN26	47.62	8.7171E-05	0.986
CAN83	62.50	5.9660E-05	0.988
CAN86	66.67	6.3238E-05	0.993
CACuO(0.50)	66.67	0.00011943	0.997
CACuO(0.25)	71.43	0.00012785	0.995
